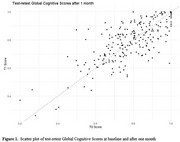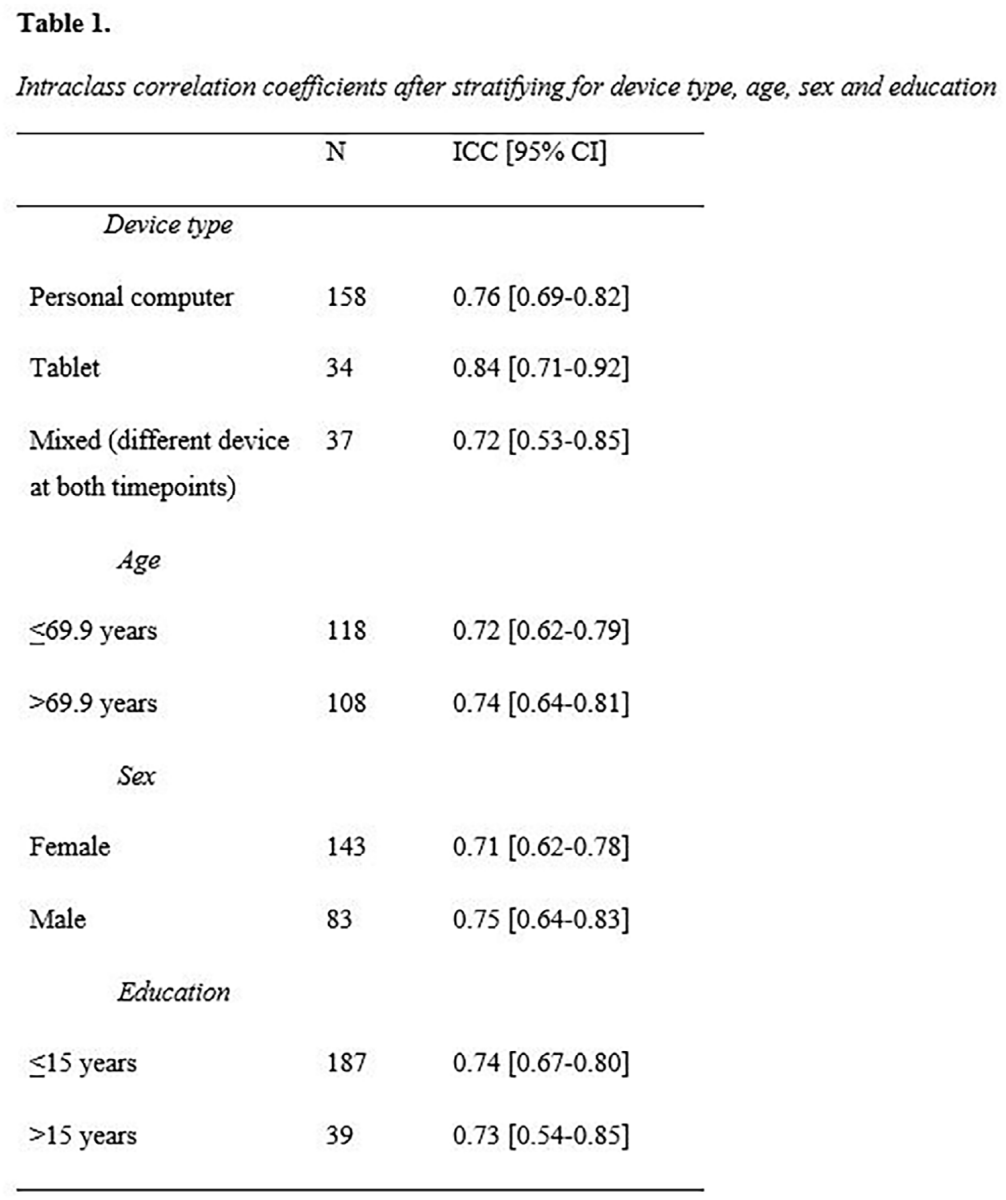# Test‐retest reliability and feasibility of web‐based cognitive assessment cCOG

**DOI:** 10.1002/alz70857_105648

**Published:** 2025-12-25

**Authors:** Sophie M. van der Landen, Sietske A.M Sikkes, Casper de Boer, Eline M.S. van Daele, Jyrki Lötjönen, Wiesje M. van der Flier, Hanneke F.M. Rhodius‐ Meester

**Affiliations:** ^1^ Alzheimer Center Amsterdam, Neurology, Vrije Universiteit Amsterdam, Amsterdam UMC location VUmc, Amsterdam, Netherlands; ^2^ Amsterdam Neuroscience, Neurodegeneration, Amsterdam, Netherlands; ^3^ Faculty of Behavioural and Movement Sciences, Department of Clinical, Neuro and Developmental Psychology, Vrije Universiteit Amsterdam, Amsterdam, Netherlands; ^4^ Combinostics Oy, Tampere, Finland; ^5^ Department of Epidemiology and Data Science, Vrije Universiteit Amsterdam, Amsterdam UMC, Amsterdam, North Holland, Netherlands; ^6^ Department of Geriatric Medicine, The Memory Clinic, Oslo University Hospital, Oslo, Norway

## Abstract

**Background:**

cCOG is a web‐based cognitive assessment that has been shown to accurately detect mild cognitive impairment and dementia. Short and accurate assessment of cognition in a remote setting is important for large‐scale disease monitoring. To this end, good test‐retest reliability is an important psychometric criterium. We examined cGOG in a sample of people at risk of dementia and determined the test‐retest reliability.

**Method:**

We included 250 participants (Mean age±Standard Deviation (SD)=69.2±7.8 years, ranged 45.6‐90.6 years, *n* = 158(63.2%) female) from the population‐based ABOARD Cohort, for whom cCOG was available at baseline (T0) and after one month (T1). Mean interval time between tests was 31.7±6.1 days. Participants completed cCOG remotely and unsupervised on their personal computer (n_T0_=180, n_T1_=189) or tablet (n_T0_=69, n_T1_=51). Most participants (80.3%) used the same device type for both tests. Completion of the five cCOG subtests, i.e. immediate and delayed recall of a word list, modified trail making A and B and fragmented letters, resulted in a global cognitive score (GCS) between 0 and 1, where a lower score reflects worse cognitive performance. Parallel versions of the word learning task were used to minimize learning effects. To determine the test‐retest reliability, we calculated the intraclass correlation coefficient (ICC) for the GCS. To explore demographic effects on the test‐retest reliability, analyses were repeated after stratifying for device type, age below versus on or above the median, sex and education below versus on or above the median.

**Result:**

237 participants (94.8%) successfully completed cCOG at baseline (mean GCS±SD = 0.79±0.15, range 0.20‐1.00) and 235 participants (94.0%) again after one month (mean GCS±SD = 0.79±0.15, range 0.26‐1.00). Individual GCS scores at both timepoints are visualized in Figure 1. We found good test‐retest reliability with an ICC of 0.75 (*n* = 226, 95%CI[0.69‐0.80], F=7, *p* <0.001). Subgroup analysis showed comparable reliability for different device types, age, sex and education groups (Table 1).

**Conclusion:**

Our findings provide support for the test‐retest reliability of cCOG for remote digital cognitive assessment in a population‐based cohort at risk of dementia. Further inspection of failed tests and outliers is needed to gain more insight in the feasibility of remote cognitive assessment.